# Changes in the Antibacterial Performance of Polymer-Based Nanocomposites Induced by Additive Manufacturing Processing

**DOI:** 10.3390/polym17020171

**Published:** 2025-01-11

**Authors:** Ana C. Pinho, Paula V. Morais, Manuel F. Pereira, Ana P. Piedade

**Affiliations:** 1 Department of Mechanical Engineering, CEMMPRE, University of Coimbra, 3030-788 Coimbra, Portugal; acspinho5@gmail.com; 2 Department of Life Sciences, CEMMPRE, University of Coimbra, 3000-456 Coimbra, Portugal; pvmorais@ci.uc.pt; 3 Instituto Superior Técnico, CERENA, University of Lisbon, Av. Rovisco Pais, 1, 1049-001 Lisboa, Portugal; mfcp@ist.ul.pt

**Keywords:** nanocomposites, additive manufacturing, air filters, antibacterial properties, micro-computer tomography

## Abstract

The idea supporting the investigation of the current manuscript was to develop customized filters for air conditioners with different pore percentages and geometry with the additional advantage of presenting antibacterial performance. This property was expected due to the reinforcement of Cu nanoparticles in the polymeric matrix of poly(lactic acid) (PLA) and polyurethane (TPU). The filaments were characterized by their chemical composition, thermal and mechanical properties, and antibacterial behavior before and after processing by fused filament fabrication. An X-ray photoelectron spectroscopy showed that the nanocomposite filaments presented Cu particles at their surface in different valence states, including Cu^0^, Cu^+^, and Cu^2+^. After processing, the metallic particles are almost absent from the surface, a result confirmed by micro-computer tomography (μ-CT) characterization. Antibacterial tests were made using solid-state diffusion tests to mimic the dry environment in air conditioner filters. The tests with the nanocomposite filaments showed that bacteria proliferation was hindered. However, no antibacterial performance could be observed after processing due to the absence of the metallic element on the surface. Nevertheless, antimicrobial performance was observed when evaluated in liquid tests. Therefore, the obtained results provide valuable indications for developing new nanocomposites that must maintain their antimicrobial activity after being processed and tested in the dry conditions of solid-state diffusion.

## 1. Introduction

The world is witnessing a constant demand for novelty, and science and technology are facing exponential growth, which can be translated into new ways to overcome daily life challenges and improve quality of life. The advent of additive manufacturing (AM), commonly known as 3D printing, is one of the pillars of this scenario. Indeed, AM opened new possibilities to bring science to our homes due to the ease of access and manipulation of the equipment for fused filament fabrication (FFF) technology, for example.

The degree of freedom to produce new parts/components/devices using different materials has made AM technologies one of the current hot topics among research groups dedicated to materials science [1]. Within a few years, researchers took giant steps, from single-material printing to multi-material structures and 4D printing, to give a few examples [2]. In addition, AM opened new venues for processing using greener techniques, in opposition to subtractive techniques, where the amount of raw material used, waste production, and energy consumption are considerably higher [3,4].

AM allows fabrication using different materials such as thermoplastic polymers, polymer-based photocurable formulations, conductive inks, and inks with biological components such as cells and composites [5]. In the last case, composite formulations usually comprise a thermoplastic or photocurable polymeric matrix and a variable filler as reinforcement depending on the chosen AM technology. In the specific case of FFF, the reinforcement does not hinder the rheological properties of the matrix; otherwise, it could make the base material impossible to print [6]. Moreover, the possibility of printing composite materials by FFF vastly increases the material range this technique can process. Also, it offers the possibility of obtaining increased properties and chemical functionalities without the need for chemical modifications that use hazardous chemicals or radiations, similar to what happened at the end of the last century [7,8,9].

Some authors have already disseminated research papers reporting the processing by FFF using a wide range of reinforcement materials such as wood [10], synthetic fibers [11,12], and metallic particles [13] in combination with printable thermoplastics. Metallic particles are particularly interesting as they may help improve the mechanical properties of thermoplastic materials processed by FFF but can also confer new features such as conductivity and antimicrobial properties [14]. The last one is of particular interest owing to the danger to consumers due to the constant increase in the resistance of microorganisms, such as bacteria, to antibiotics and other associated therapies [15].

It is well established in the literature that some metallic and metallic oxide particles, such as copper (Cu), silver (Ag), or zinc oxide (ZnO), to mention a few, have antimicrobial properties [16,17,18]. Also, some studies show that the presence of a bimetallic (Ag-Au) nanoparticle helps increase the antibacterial activity of silver [19]. As expected, two metallic elements will act as galvanic cells in the growth medium, thus enhancing the oxidation of the less stable element—silver. This will increase the rate of the formation/release of Ag ions and, consequently, the antibacterial activity.

Moreover, many composite materials using such particles as reinforcement have already been investigated for the biomedical field and medical facilities [20,21] and other kinds of environments, such as in space [22]. Most of the reported work describes the synthesis and characterization of those materials, where their availability on the surface of the nanocomposites enables and facilitates the diffusion of the metallic material and, consequently, the antibacterial activity [23].

With the evolution in FFF and the significant increase in the demand for antimicrobial and antiviral devices triggered by the late pandemic, researchers explored the possibility of printing parts with antimicrobial properties from composite materials with antimicrobial fillers [24]. In work conducted by Brounstein et al., poly(lactic acid) (PLA)-based composite filaments with TiO_2_ and ZnO ceramic particles as reinforcement were produced, and their antimicrobial performance was evaluated [25]. In addition, poly(ethylene glycol) (PEG) was added to the formulations to enhance mechanical performance. The results show that incorporating the ceramic particles reduced the PLA pitting caused by microbial activity, and incorporating PEG did not hinder such behavior.

Following a similar approach but using metallic nanoparticles, Podstawczyk et al. describe the preparation and evaluation of the antimicrobial performance of PLA filaments with silver nanoparticles [26]. Herein, the printed parts proved to be effective against two different bacterial strains. However, as in most of the published literature on this approach, the antimicrobial results appeared only after 24 h of incubation of the printed parts in an aqueous solution. However, these tests do not mimic the real conditions in applications where the contact is shorter and occurs in a dry medium, such as in air conditioner systems.

Concomitantly, and in another area, it has to be considered that within building premises, air conditioning systems are responsible for a third of all the embodied carbon used, potentially making them a particularly pertinent case of construction waste. Moreover, these conditioning systems are also responsible for severe bacterial infections [27]. Because some research groups are working on air conditioning systems that are entirely 3D printed from recyclable thermoplastic polymers and also fully recyclable at the end of the life cycle [28], an important advancement in the knowledge is to study 3D-printed filters, customizable for each air conditioner, in a recyclable thermoplastic polymer that simultaneously presents antimicrobial properties.

This work also intends to show that “plastics” can efficiently contribute to decreasing the carbon footprint and related consequences. However, processing studies for AM systems are always a critical topic of ongoing study [29] and justify the need to evaluate the effect of processing parameters on the selected properties, such as in the case of the antibacterial ones.

The present work intends to develop customized membrane filters produced by FFF (Fused Filament Fabrication) with antibacterial properties. The thermoplastic-based composites used were filaments of PLA and TPU with Cu (1 wt.%) reinforcement (PLA_Cu and TPU_Cu) that were characterized by several techniques before and after AM processing. The chosen concentration of the metallic reinforcement was made considering the balance between the envisioned antibacterial activity, which increases with the increased concentration of the metallic materials [30], and the cytotoxicity that high concentrations present to eukaryotic cells [31]. Due to the envisaged application, the antibacterial performance of the filaments and the 3D-printed filters were studied against three different bacterial strains, including Gram-positive (Gram+) and Gram-negative (Gram−).

## 2. Materials and Methods

### 2.1. Materials

In the present work, two classes of materials were considered, namely thermoplastic polymers and thermoplastic polymer-based nanocomposites reinforced with copper nanoparticles. The nanocomposite filaments had 1% wt. of Cu, and the thermoplastic PLA and TPU, with no antibacterial activity, were used as controls. All the filaments, with a diameter of 1.75 ± 0.03 mm, were purchased from Filament2Print (Pontevedra, Spain) and used without further modifications.

### 2.2. Processing by Additive Manufacturing

The filters were manufactured by FFF using the FlashForge^TM^ Creator 3 equipment equipped with a 0.4 mm diameter nozzle. All the specimens were cylinder-shaped with a 10 mm diameter and thickness of 2 mm. PLA and PLA_Cu were processed with a printing temperature of 220 °C, bed temperature of 50 °C, and 50 mm⋅s^−1^ printing speed. In turn, TPU and TPU_Cu were printed at 220 °C and 25 mm·s^−1^ on a bed at 70 °C. The processing parameters were selected according to the thermal and mechanical characterizations of the filaments, as described in the following subsections.

**Figure 1:**
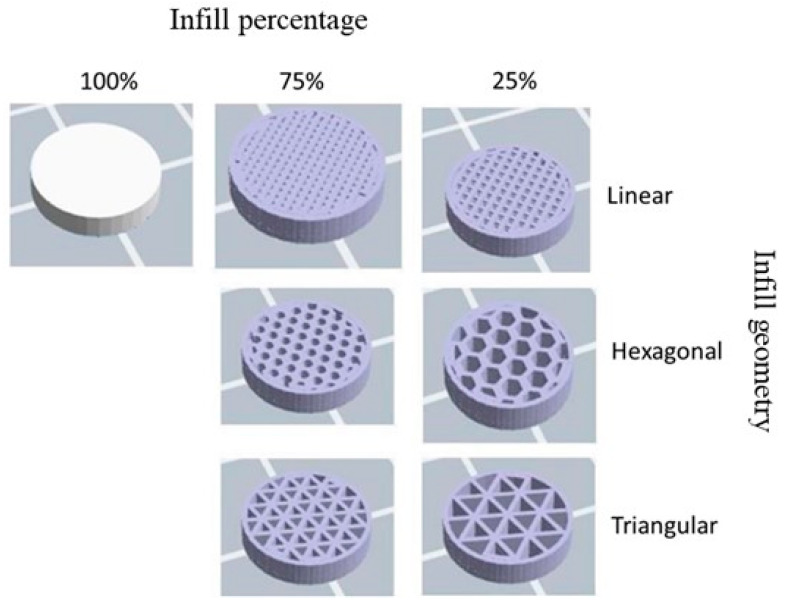
Design of the specimens with different infill geometry and percentages. Images were obtained from the *gcode* files after being sliced in the FlashPrint 5.0 software.

Moreover, the optimization of the processing parameters also results from an accumulated experience of more than five years in the research group. Three infill patterns were printed for each material: linear, hexagonal, and triangular (Figure 1). In addition, the infill percentage also varied. Three percentages were considered for the linear infill samples: 100%, 75%, and 25%. Only 75% and 25% infill percentages were studied for the hexagonal and triangular patterns since 100% infill is independent of the geometry. These were the variable parameters that aimed at studying the influence of the percentage and shape of the porosity on the potential antibacterial activity of the filters, as the intended application was the processing of completely customized filters.

### 2.3. Chemical and Morphological Characterization

For control purposes, the chemical structure of the commercial filaments was assessed by Fourier-transform infrared spectroscopy (FTIR) using the Frontier model equipment from PerkinElmer (Madrid, Spain) equipped with an attenuated total reflectance (ATR) module with a diamond/ZnSe crystal, FR-DTGS detector, and a KBr beam splitter. The collected spectra were analyzed using the SPECTRUM 10 STD software. All the analyses were conducted at 20 °C, with a 4 cm^−1^ resolution, at a constant force of 80 N, and 16 accumulation interferograms.

An X-ray photon spectroscopy (XPS) further investigated the composite filaments’ chemical composition. The equipment used was an Axis Ultra Has from Kratos (Tokyo, Japan), with an incident monochromatic radiation Al-Kα (λ = 0.83401 nm), following the procedure reported elsewhere [32].

The morphology of the nanocomposite filaments and printed specimens were studied by scanning electron microscopy (SEM) using the MERLIN61-50 equipment from ZEISS (Dresden, Germany) equipped with an energy-dispersive X-ray spectrometer (EDS) for chemical elemental analysis with an acceleration voltage between 1 and 5 kV. Due to the non-conductive electric nature of the polymeric materials and composites, the samples were sputter-coated with a 5 nm thin film of gold before characterization.

### 2.4. Thermal Characterization

The thermal stability of the nanocomposite filaments was studied through a thermogravimetric analysis (TGA) using the Q500 V20.14 equipment from TA Instruments (Porto Portugal). The temperature range was 25–600 °C, with a heating rate of 10 °C·min^−1^ under a constant nitrogen flux of 50 mL·min^−1^. The obtained data were processed using TA Instruments Universal Analysis 2000 provided by the supplier.

Differential scanning calorimetry (DSC) analysis was used to assess the thermal events of the filaments and printed specimens. The equipment used in the present work was a DSC Q100 V9.9 from TA Instruments. The tests were conducted under a constant nitrogen flux of 50 mL·min^−1^ with a heating rate of 10 °C·min^−1^. The essay temperature ranged between −80 °C and 300 °C.

### 2.5. Mechanical Characterization

Tensile tests of the filaments were conducted to provide information useful to define the printing speed as the materials experience tensile forces while being extruded through the printer nozzle. A Shimadzu Autograph AGS-X equipment (Tokyo, Japan) with a 5 kN load cell and a 2 mm⋅min^−1^ grip speed was used at room temperature. Five 100 mm long samples were tested for each material, and a distance between grips (L_0_) of 50–65 mm was kept constant.

### 2.6. Micro-Computer Tomography Imaging

This research used micro-computer tomography (µ-CT) to study the volumetric distribution of Cu within the polymeric matrix for filaments and printed specimens. All the samples were scanned using the μ-XCT Skyscan 1172 microtomograph (Madrid, Spain) by rotating the sample over 180° with a variable rotation step of 0.20–0.30° depending on the selected resolution and according to the sample size. The acquisition conditions were optimized to achieve the best image contrast. The acquired data were then processed and analyzed using NRecon^®^1.6.3 routine, volumetric visualization was achieved with the DataView^®^ and CTvox^®^ programs by Bruker, and the pixel intensity was determined using ImageJ 8.0. Table 1 lists some of the parameters used during the scanning procedure.

### 2.7. Antibacterial Tests

All the tested specimens were sterilized with 2 mL ethanol solution (70% *v*/*v*) under soft stirring (50 rpm) for 10 min and left to dry in air for 20 min. The overall procedure was performed in a hot flow.

The antibacterial activity of the filaments and printed specimens was determined by observing the inhibitory halo formation in a solid medium. For the printed specimens, tests in a liquid medium were also performed. For the solid medium tests, with the pre-inoculations of *Pseudomonas aeruginosa* (*P. aeruginosa*), *Escherichia coli (E. coli*), and *Staphylococcus aureus (S. aureus*), a suspension of 0.85% (*w*/*v*) NaCl solution was made with an optical density (OD) of OD = 0.5 on the McFarland scale.

From each solution, 100 μL were inoculated in Petri dishes with a solid Luria–Bertani (LB) medium previously sterilized. The surfaces under study were placed in contact with the inoculated medium. The Petri dishes were incubated for 24 h at 37 °C. After this period, the dishes were observed to assess whether or not the inhibition halo existed. The solid LB medium was constituted by the following reagents (weight per 100 mL of the final volume): bacteriological agar (1.5 g), NaCl (1.0 g), yeast extract (0.5 g), and tryptone (1.0 g).

For the liquid tests, the same bacterial strains were used to prepare suspensions in liquid media (R2A liquid), with 0.5 turbidity units according to the scale of McFarland and incubated at 37 °C under 136 rpm. After 24 h, the suspensions were diluted to an optical density equal to 0.2, and 2 mL of these suspensions were placed in the multiwall plates, and the filaments and printed specimens were completely submersed with the suspensions. After 38 h, the optical density was measured: the lower the values, the higher the antibacterial activity. The tests were performed in duplicate and repeated three times.

## 3. Results and Discussion

### 3.1. Preliminary Characterization of the Filaments

**Figure 2:**
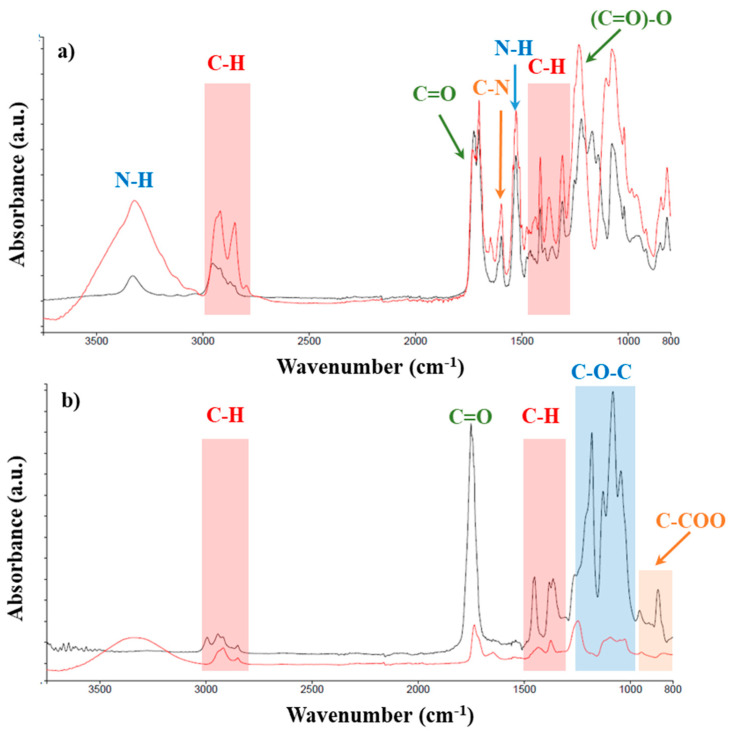
FTIR spectra of the commercial filaments: (**a**) TPU (black) and TPU_Cu (red); (**b**) PLA (black) and PLA_Cu (red).

All the filaments were analyzed by FTIR regarding their chemical composition due to the need for more information than the one provided by the supplier concerning the possible incorporation of additives (Figure 2). The characterization showed that in all the filaments, the signature bands of their base polymers (PLA and TPU) were present [33]. In addition, no unexpected bands were observed for any of the materials.

**Figure 3:**
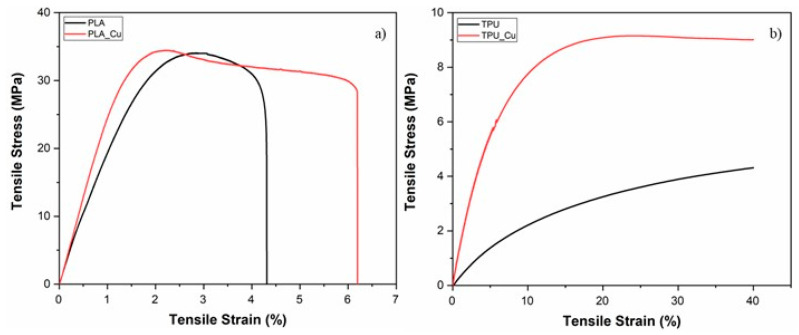
Representative stress–strain curves from the tensile tests of the filaments: (**a**) PLA (black) and PLA_Cu (red); (**b**) TPU (black) and TPU_Cu (red).

Also, to establish if the presence of the metallic material in the composite could influence the printing parameters, namely the printing speed, tensile tests were performed on the materials in filament form (Figure 3). This correlation is significant in this study because FFF technology subjects the filaments to tensile forces during the extrusion through the nozzle.

Additionally, the mechanical tests allowed us to compare the influence of the metallic reinforcing particles in the tensile stress (σ), strain at break (ε), and Young modulus (E) of the materials (Table 2).

As expected, introducing the copper particles increases the mechanical properties of the filaments. This increase is less evident for the PLA-based composite due to the more brittle nature of the base thermoplastic polymer, as observed by other authors [34]. The TPU-based nanocomposite shows an increase in all the evaluated properties: ε increased by 125% and E by around 330%. This behavior was also reported by other authors [35] and is due to a more ductile behavior of TPU when compared with PLA.

Several reported mechanisms try to explain the increase in all mechanical properties, evaluated by tensile tests, by incorporating nanoparticles. Some authors state that the incorporation of copper nanoparticles could promote the interconnection of hard elements forming paths, producing a change in the distribution of the compound, which translates into an increase in the toughness of the material [36,37]. Others attribute the performance to good homogeneity, processing conditions, and interactions between the nanoparticles and matrix, which allowed the transfer of efforts of the matrix to the nanoparticles [38].

The experimental results indicated that the same printing speed could be used within each pair of materials, PLA/PLA_Cu and TPU/TPU_Cu.

**Figure 4:**
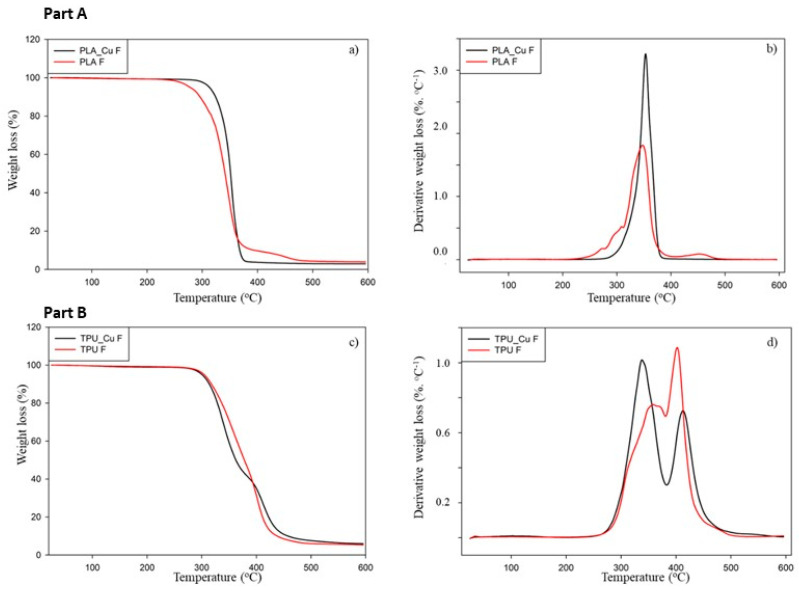
Part A—(**a**) thermogravimetric curves of the filaments PLA and PLA_Cu and (**b**) respective derivative curves; Part B—(**c**) thermogravimetric curves of the filaments TPU and TPU_Cu and (**d**) respective derivative curves.

Before any processing step using commercial polymeric filaments, it is also important to evaluate their thermal stability and investigate the temperature range in which their thermal events occur to select the printing temperatures correctly. Furthermore, in the present work, the effect of incorporating Cu nanoparticles in the thermal behavior of the filaments also needed to be assessed. For this reason, the thermal behavior of PLA, PLA_Cu, TPU, and TPU_Cu commercial filaments was investigated using TGA and DSC analysis. Figure 4 plots the TG and DTG curves obtained for the nanocomposite commercial filaments and for non-reinforced matrix.

The obtained results show that all the filaments are thermally stable until ca. 250 °C. Regarding the PLA filaments, both profiles are not entirely similar because PLA presents two decomposition stages, while PLA_Cu displays only one. It is well established that pure PLA only presents one decomposition step between 300 and 400 °C, approximately [39]. Suppliers do not provide information concerning the molecular weight of the commercial filaments, but in the case of PLA, the length of the polymeric chains is not very uniform. Consequently, shorter chains decompose at 250 °C, while most of the polymeric network starts to degrade above 300 °C.

In turn, the PLA_Cu filaments exhibit a decomposition profile with a smaller width of the DTG curve, indicating that the molecular weight distribution is much narrower; thus, most of the polymeric chains degrade at the same temperature range. By the end of the essay, the residual char is below 1% for both filaments, confirming that the Cu content is relatively low according to the manufacturer’s information.

On the other hand, both the TPU and TPU_Cu filaments display two well-defined decomposition steps. The first one, between 260 and 330 °C, is assigned to the degradation of the hard segments of TPU owing to the presence of urethane bonds [40]. In turn, the second step (380–500 °C) corresponds to the degradation of the polyol chains of the soft segments of the TPU structure [41]. The molecular weight distribution of TPU_Cu also seems to be narrower than that of TPU, as the DTG curves present a thinner width than TPU. The residual char observed at 600 °C is around 6% in both the TPU-based filaments, thus confirming that, once again, the Cu content in the TPU filament is low.

To sum up, the thermogravimetric analysis proved that incorporating Cu did not significantly alter the thermal stability of PLA and TPU. As expected, the thermogravimetric curves of the printed specimens using the PLA_Cu and TPU_Cu filaments were quite similar to those of the base materials, which indicates that 3D printing processing does not influence the thermal stability of the composite materials.

Continuing in the thermal characterization, but focusing on the characteristic temperatures, a DSC analysis was performed. The results showed that the presence of Cu did not significantly alter the characteristic thermal transition temperatures of PLA and TPU, namely *T_g_* = glass transition temperature, *T_cc_* = cold crystallization temperature, and *T_m_* = melting temperature. Indeed, all the events that can be identified for the pristine materials can also be seen in the composite filaments. However, a slight temperature difference was observed, which might result from the different average molecular weights (as shown in the TGA) of each filament since it directly affects the mobility of the polymeric chains. The characteristics temperatures determined by DSC are summarized in Table 3.

The PLA-based filaments and printed specimens display three different thermal transitions. The first corresponds to the glass transition temperature (*T_g_*). The second event is exothermic and refers to the cold crystallization temperature (*T_cc_*). Finally, the last event, occurring at the highest temperature, is the melting temperature (*T_m_*). These results agree with the literature, in which the three transitions of PLA are well documented [42,43].

After determining each event temperature, it is possible to infer that neither the incorporation of Cu nor the processing by 3D printing significantly influences the temperature at which each thermal event occurs. Only *T_m_* in the commercial filament stands out, showing the highest temperature. However, as aforementioned, such a fact can be related to the molecular weight of the filament. Regarding the nanocomposites, no relevant dissimilarities were found.

Within a similar behavior already observed for the PLA-based materials, all the TPU samples show the same thermal events at almost the same temperatures, and the determined temperatures are according to the available literature [44].

The data collected from DSC prove that all the samples have semi-crystalline structures, as they all present both glass transition and melting temperatures. In addition, incorporating Cu particles did not alter the mobility of the polymeric chains. Moreover, the thermal characterization of the printed samples validated the selection of the experimental printing parameters as the optimized ones for the polymer composites.

### 3.2. Morphological and Chemical Characterization

**Figure 5:**
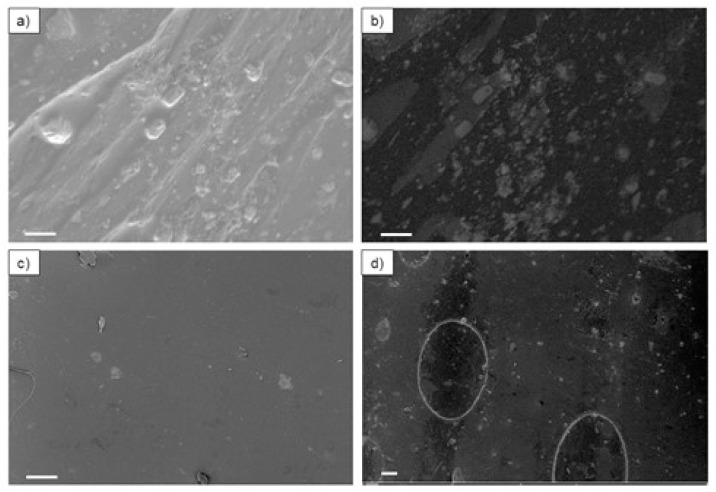
SEM micrographs of PLA_Cu (**a**,**b**) and TPU_Cu (**c**,**d**). Micrographs (**a**,**c**) correspond to the images obtained in the secondary electron mode; (**b**,**d**) correspond to the backscattered electrons mode. Scale bar (**a**,**b**) = 1 μm; (**c**) = 10 μm; (**d**) = 20 μm.

According to the supplier, the composite commercial filaments PLA_Cu and TPU_Cu have antimicrobial properties due to the Cu nanoparticles in the polymer matrix phase. In the literature, despite the uncertainty of the actuation mechanisms, it is well established that some Cu ions have antimicrobial activity [45]. The activity of different valence of the copper ions depends on several factors, such as particle size, shape, dry or wet environment, and exposure time [46]. Therefore, for Cu nanoparticles to be effective against pathogens, they must be located at the surface of the materials to enable their interaction with the prokaryotic cells [47]. For this reason, the morphology of the as-received commercial filaments was investigated by SEM, and the resulting micrographs are presented in Figure 5.

From the observation of the micrographs, it can be stated that the morphology of PLA_Cu and TPU_Cu is quite similar. Indeed, in both materials, the presence of two different phases at the surface can be observed: the continuous polymeric phase and the reinforcement in the form of particles, as expected.

However, particles are not uniformly distributed within the surface of the filament. Moreover, contrary to what the supplier advertises, these particles (represented in lighter gray) do not present nanometric dimensions. This might have occurred due to a possible agglomeration of the particles while manufacturing the filament, as reported by other authors [48], due to the great dielectric constant of the metallic nanoparticles, which favors the attraction between them.

**Figure 6:**
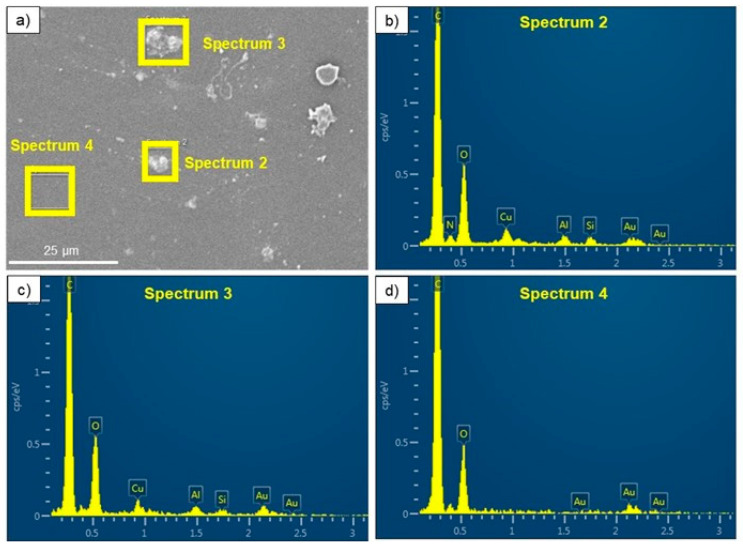
(**a**) Representative micrograph of the TPU_Cu filament, with the yellow squares indicating the areas analyzed by EDS. Spectra 2 and 3 (**b**,**c**), respectively, show the presence of Cu particles; (**d**) spectrum 4 shows the absence of metallic material in the matrix.

As a result, the observed particles are in the micrometric and submicrometer range. A qualitative chemical elemental analysis confirmed the chemical elements in both phases. Due to the similarity of the materials’ surfaces, Figure 6 shows only the representative plots of TPU_Cu.

**Figure 7:**
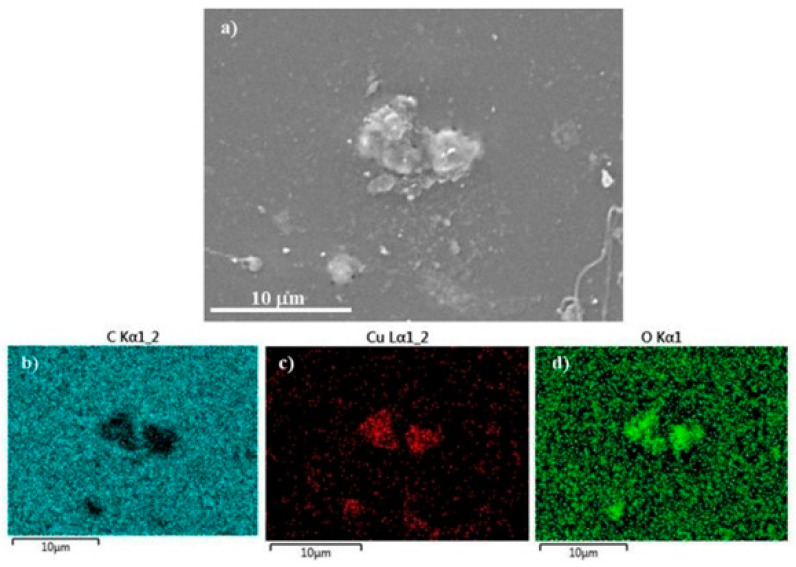
Elemental map distribution of the surface of TPU_Cu: (**a**) representative SEM micrograph; (**b**) carbon distribution map; (**c**) Cu distribution map; (**d**) oxygen distribution map.

The qualitative analysis of the chemical elements present on the surface of both samples indicates the presence of Cu, thus reinforcing the idea that the lighter gray particles in Figure 6 correspond to Cu particles. The elemental distribution maps obtained from the EDS analysis (Figure 7) also confirmed this assumption.

The elemental distribution maps revealed that the observed particles corresponded to Cu particles. In addition to Cu, these lighter gray regions also present high oxygen concentrations, which can be due to the possible oxidation of the Cu particles.

**Figure 8:**
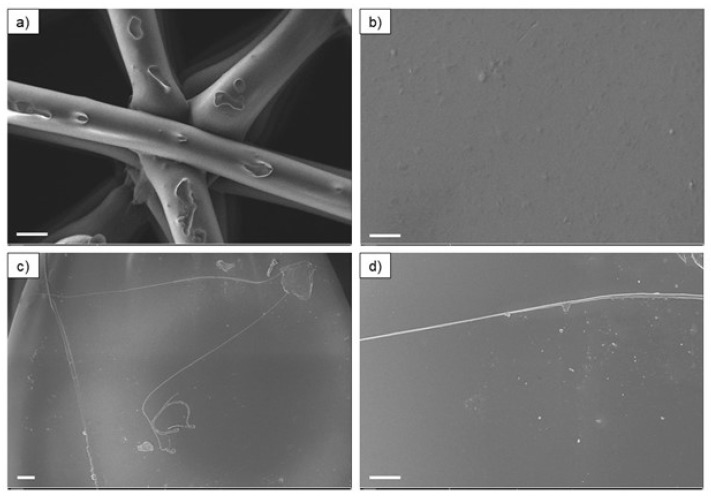
SEM micrographs of 3D-printed filters of PLA_Cu (**a**,**b**), and TPU_Cu (**c**,**d**). Scale bar: (**a**) = 200 μm; (**b**) = 1 μm; (**c**) = 20 μm; (**d**) = 10 μm.

**Figure 9:**
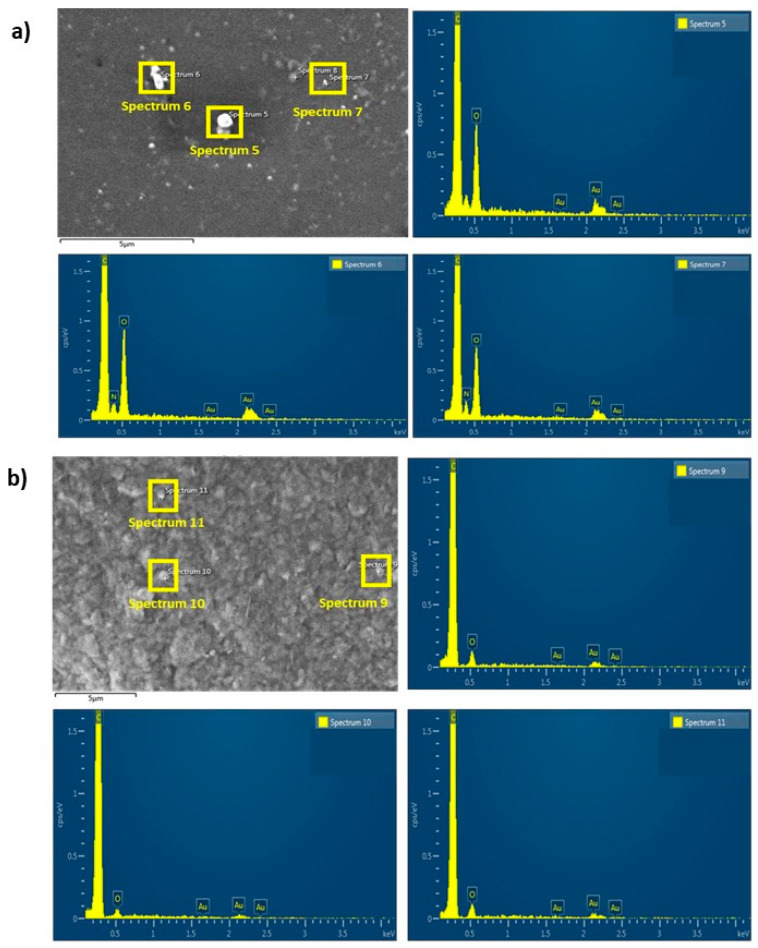
SEM representative micrograph of the printed specimen and EDS qualitative spectra of the yellow selected areas in the micrograph: (**a**) TPU_Cu; (**b**) PLA_Cu. All the spectra show the absence of Cu, either in the matrix or in the particles.

The 3D-printed filters also underwent morphological study by SEM, and the micrographs are presented in Figure 8. EDS also evaluated the qualitative spectra of the chemical elements on the surface of the printed samples, and the results are shown in Figure 9.

No significant differences were observed between the micrographs of the printed specimens of PLA_Cu and TPU_Cu. The previously observed particles at the surface of the filaments are no longer visible. Such a fact suggests that the Cu particles, regardless of their size, have been “buried” in the polymeric material due to the processing by AM. Indeed, such behavior may have been favored by the printing temperature achieved since it is higher than the material’s melting temperatures.

The analysis of all the qualitative spectra shows that Cu particles are not on the surface of the printed filters, as no detection of this element was observed. Indeed, the spectra resemble those previously obtained from the areas surrounding the Cu particles, i.e., from the polymeric matrix.

The difference between the surface energy of the different classes of materials, polymer and metal, in the nanocomposite may justify these observations. Metals and ceramics, considering that some of the copper is oxidized, have much higher surface energy than polymers. The surface energy for bulk copper is around 1400 mJ·m^−2^ [49], while for PLA and TPU, this value is circa 35 and 45 mJ·m^−2^, respectively [50,51]. Therefore, the cohesion forces between the reinforcement particles are higher than the adhesion forces with the polymeric matrix. Consequently, the particles tend to agglomerate and, during manufacturing, are buried into the polymer matrix. Accordingly, the composite will present a surface with the lowest possible surface energy (only polymer), as most materials minimize their surface energy and produce characteristic equilibrium shapes determined by Wullf’s theorem [52].

**Figure 10:**
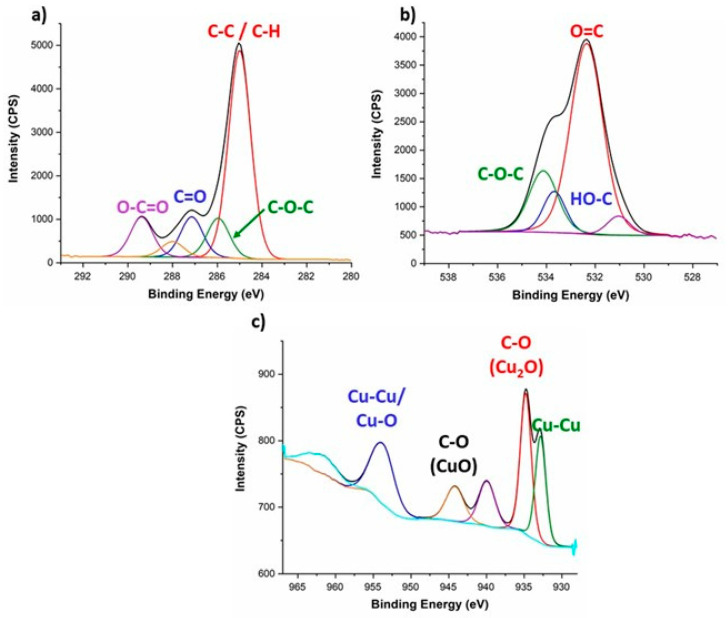
Deconvolution of representative XPS high-resolution spectra of PLA_Cu for (**a**) C 1s, (**b**) O 1s, and (**c**) Cu 2p.

The surfaces were characterized by XPS to clarify the chemical state of the copper particles in the filaments and to confirm the absence of copper in the printed filters. Moreover, the EDS elemental analysis of PLA_Cu and TPU_Cu revealed the presence of oxygen (O), which could be related to the oxidation of the Cu particles. In the context of the envisaged application, the oxidation of elemental Cu into CuO or Cu_2_O would be of main interest since this oxide is known to attain enhanced antimicrobial activity [53]. To clarify this point, an XPS analysis was performed in both the PLA_Cu and TPU_Cu filaments and printed specimens. Figure 10 plots the deconvolution of the PLA_Cu high-resolution spectra.

The results obtained for PLA_Cu and TPU_Cu were quite similar. In both cases, the analysis allowed us to conclude that the Cu particles are at the surface of the filament, which corroborates the previous EDS analysis. The deconvolution of the high-resolution spectra and identification of the XPS peaks [54] revealed the expected PLA bonds, C-C, C-H, C-OH, O-C=O, and C=O. In turn, the Cu 2p spectrum shows the presence of oxides, Cu_2_O, CuO, and metallic Cu, corresponding to the Cu^+^, Cu^2+^, and Cu^0^ oxidation states of copper, respectively. These oxidation states occur due to the constant oxidation state of oxygen (O^2−^), and the resulting compounds are electrically neutral. Due to the presence of metallic ions, the composite filaments are expected to present some antimicrobial properties [55].

**Figure 11:**
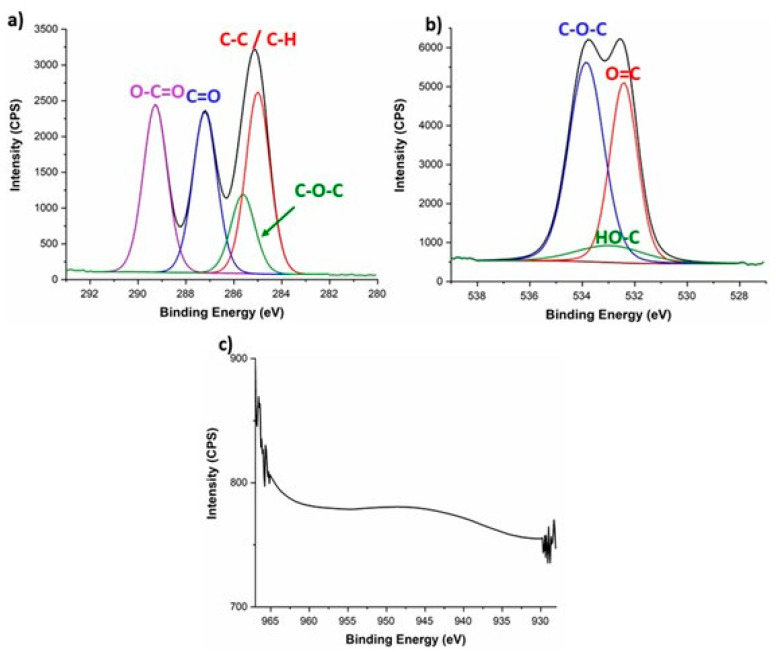
High-resolution XPS spectra deconvolution of printed PLA_Cu for (**a**) C 1s, (**b**) O 1s, and (**c**) Cu 2p.

After analyzing the composite filaments, XPS also investigated the chemical composition of the printed specimens of PLA_Cu and TPU_Cu. The representative deconvolution of the high-resolution XPS spectra obtained for the PLA_Cu printed samples is displayed in Figure 11.

Despite the differences in peak intensity, the C 1s and O 1s profiles obtained for the printed specimens resemble the ones previously discussed for the filaments. No alterations in the PLA chemical composition were expected since the 3D printing technology used is based on temperature, which does not influence the chemical components at the chemical bond level. Indeed, the temperature-based extrusion associated with FFF only influences the movement and alignment of the polymeric chains since it is below the degradation temperature (see Section 3.2).

Moreover, from what concerns the Cu content, it can be observed that no peaks are presented for the binding energy range associated with this element. SEM micrographs have previously shown that these particles disappear from the surface once the material is printed. Therefore, it is confirmed that the metallic particles at the filament’s surface migrate during the manufacturing of the components.

**Figure 12:**
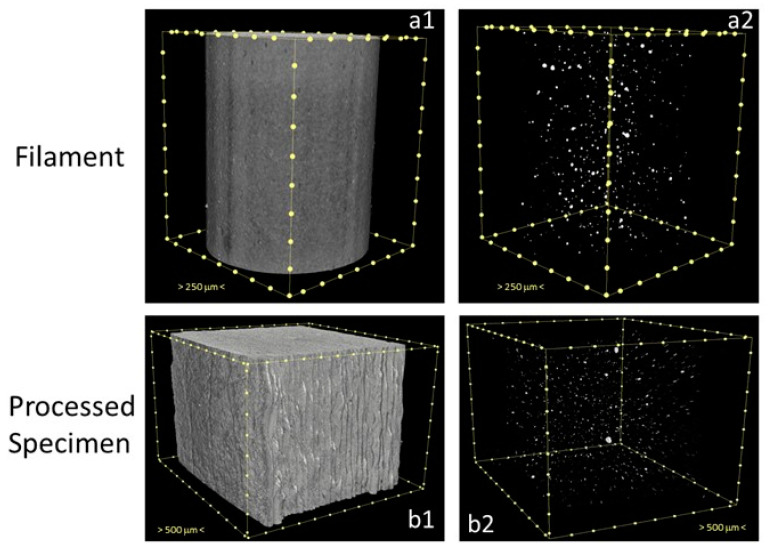
μ–CT images of the PLA_Cu filament (**a1**) showing the homogeneous distribution of the Cu particles (**a2**); after processing (**b1**), the Cu particles are mainly present in the interior of the printed specimen (**b2**).

The μ-CT images further confirmed these results in Figure 12 for PLA_Cu, which also allows the confirmation of the micron and submicron dimensions of the metallic Cu particles. It is clear that in the filament (Figure 12(a1)), the distribution of the metallic particles (Figure 12(a2)) is uniform on the surface and in the interior of the composite. In contrast, in the printed sample (Figure 12(b1)), the presence of Cu particles is mainly on the interior of the processed material (Figure 12(b2)).

### 3.3. Antibacterial Tests

As aforementioned, the produced specimens were printed using different infill patterns and densities to study the influence of the pore size geometry on the antibacterial activity of 3D-printed filters. For control purposes, specimens with 100% infill and no geometrical pores were also considered in the essay. The antimicrobial properties were assessed by contact in solid media for further observation of the inhibition halo formation. Three strains of bacteria were used where *P. aeruginosa* and *E. coli* (Gram−) are rod-shaped, while *S. aureus* (Gram+) presents a spherical shape.

**Figure 13:**
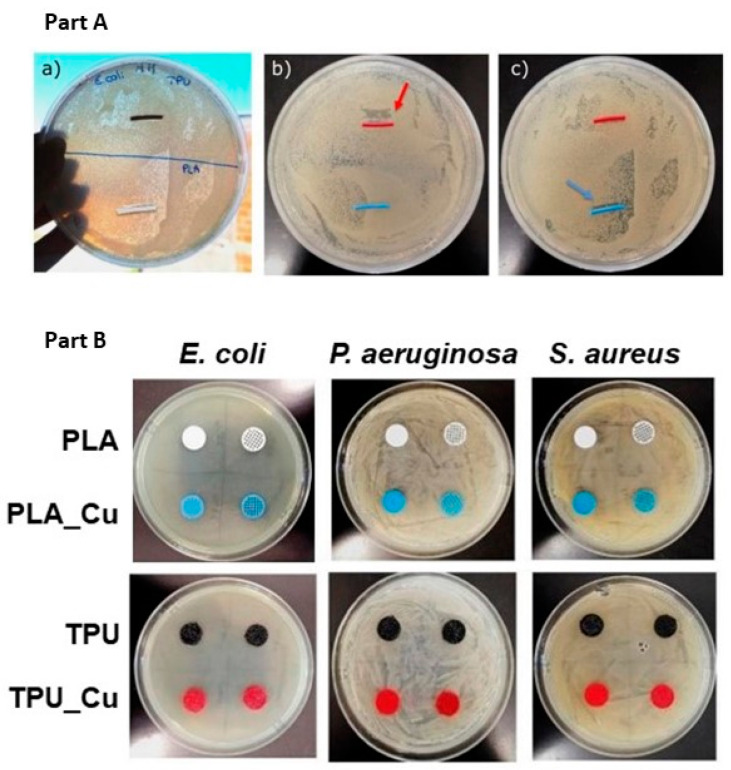
Part A—representative macrographs of the inhibition halo formation of the filaments (**a**) PLA (white) and TPU (black); (**b**,**c**) PLA_Cu (blue) and TPU_Cu (red) with *E. coli*. The arrows (red in (**b**) and blue in (**c**)) point to the places where no bacterial growth was observed. Part B—representative images of the inhibition halo formation tests of some printed filters with the three bacterial strains.

The antimicrobial tests began by evaluating the performance of the commercial filaments as received. Despite the bacterial strain used, the results obtained for the commercial filament were quite similar. For that reason, a representative image for each case is displayed in Figure 13-Part A for the test with *E. coli*.

For comparative purposes, the antimicrobial study included the PLA and TPU filaments with no Cu particles. As expected, no inhibition halo was observed for either of these materials since PLA and TPU are not antibacterial polymers [56].

PLA_Cu and TPU_Cu showed that no inhibition halo formed around the filaments. Nonetheless, when the filaments were rolled to the side, it was observed that no bacteria had grown in the area under the filaments. Therefore, the filaments presented antibacterial properties as advertised by the manufacturer. The small contact area between the filament and the solid media can explain the lack of a macroscopic visible inhibition halo.

The antimicrobial tests were also performed using the printed parts from the composite filaments with dissimilar infill patterns and densities. Figure 13-Part B presents a set of representative macrographs of the studied samples.

Considering all the previous characterizations, the lack of antibacterial activity of the printed filters when tested in a solid medium was expected. If the antibacterial material (copper and its oxides) are absent from the surface, PLA and TPU alone cannot inhibit bacterial growth. Moreover, because the tests were conducted in a solid medium, the diffusion of the metallic ions from the inner portion of the printed filters to the surface occurred at a low rate, which did not allow for the inhibition halos to form during the duration of the tests. Diffusion implies the constant movement of atoms or molecules. This process occurs in all the phases of matter but at different speeds. Despite the crystalline or amorphous structure, the thermal vibrations allow limited but significant movement of atoms in solids. Nonetheless, time is fundamental for diffusion to occur and its consequences to be visible [56].

When tested in a liquid medium, the results were different, and all the samples presented some degree of antibacterial properties according to the lower optical density values when compared with the controls (PLA and TPU without Cu) presented in Table 4. The diffusion of the copper ions occurs at a higher speed due to electric potential at the solid–liquid interface, which is the driving force for transporting electrical charges in a homogeneous solid [56].

The lower the infill percentage, the higher the antibacterial performance is due to the large surface area of contact with the bacterial suspension. Moreover, the simultaneous presence of Cu-Cu and Cu-O bonds are propitious to induce bacterial death, where the Cu-O generates electron-gap pairs by photoabsorption, and Cu absorbs photo-excited electrons, separating peers and leading to greater efficiency in the photo-inactivation process of bacteria [57].

The results in the solid medium contradict those reported in the literature [58,59,60], where 3D-printed PLA-based composites with Cu or Ag present antibacterial activity. The major difference is that in those studies, the tests are always performed by counting the number of colony-forming units (CFU) after the printed specimens have been in contact with a liquid medium for at least 24 h [61]. During the immersion time, Fick’s law occurs and the metallic ions can diffuse from the polymer into the liquid medium used to grow the bacteria, as shown by the liquid tests within this study.

Although antibacterial properties were obtained in the liquid tests, this type of test does not mimic the conditions for real applications intended in this work. The printed filters were intended to be used in dry environments, such as air conditioners, to prevent bacterial contamination of indoor spaces. Consequently, the only valuable test that mimics such conditions is the growth inhibition halo.

## 4. Conclusions

The present study aimed to produce filters for devices, such as air conditioners, by 3D printing thermoplastic-based composites (Cu-reinforced) to allow customization, antimicrobial performance, recyclability, and the easy replacement of broken parts. Therefore, it was imperative to evaluate the effect of the processing parameters on the antimicrobial activity of the 3D-printed Cu-reinforced thermoplastic nanocomposite, namely PLA_Cu and TPU_Cu, and compare them with the properties before processing.

The characterization of the commercial filaments involved chemical, thermal, and mechanical properties. The presence of the metallic particles at the surface of the commercial filaments was confirmed by SEM-EDS and XPS. The deconvolution of high-resolution XPS spectra revealed that copper was present in different valence states, including Cu^0^, Cu^+^, and Cu^2+^, indicating a mixture of metallic and oxidized copper.

After 3D printing, no copper was detected at the surface of the filters, regardless of the pore geometry or infill density. The assumption that Cu particles agglomerated in the interior of each deposited layer during processing and were no longer present in any surface of the printed filters suggested by SEM was confirmed by μ-CT.

When evaluated by a solid-state diffusion test, the nanocomposite filaments presented antimicrobial activity against three different bacterial strains. Nonetheless, using the same approach, none of the 3D-printed filters led to the formation of an inhibition halo with the same bacterial strains. These results were expected after the chemical characterization of the composites. Considering that metal gives the antibacterial performance, more specifically the metallic ions, and as these particles were not present on the surface of the 3D-printed filters, no inhibition of bacterial growth was expected.

However, the antibacterial properties were observed in the liquid tests with better results for the lower infill percentages without significant differences concerning the used geometry. These results indicate that although the solid-state tests were performed in dry conditions (to mimic what happens in air conditioners), the test duration was not enough to allow the diffusion of the metallic ions from the inner part of the filters to their surface.

The present study highlights the need for further research in the area of nanocomposite thermoplastic polymers that present antibacterial properties in short-time tests that mimic dry conditions.

## Figures and Tables

**Table 1:** Summary of the parameters used in the scanning procedure.

Parameters	
Pixel size	2.5–3.8 µm
Filament Current	113–131 µA
Voltage	70–85 kV
Power	10 W
Exposure time	1900–2100 ms
Number of Images	737–1105
File Type	16-bit
Scan Duration	2 h 30–3 h 00

**Table 2:** Mechanical properties of the filaments obtained from the tensile tests.

Filaments	Mechanical Properties		
	σ (MPa)	ε (%)	E (MPa)
PLA	33.8 ± 3.0	4.4 ± 0.4	15.1 ± 2.0
PLA_Cu	34.1 ± 3.3	6.2 ± 0.5	18.3 ± 2.2
TPU	4.0 ± 0.8	-- *	0.24 ± 0.1
TPU_Cu	9.0 ± 1.3	-- *	0.80 ± 0.2

* the filaments did not break because the maximum elongation was attained.

**Table 3:** Summary of the determined characteristics temperatures of the PLA and TPU-based filaments (F) and printed (P) specimens.

Material	*T*_*g*1_ (°C)	*T*_*g*2_ (°C)	*T_cc_* (°C)	*T_m_* (°C)
PLA F	59.8	-	98.4	171.1
PLA_Cu F	59.7	-	103.6	150.9
PLA_Cu P	54.7	-	109.5	152.4
TPU F	−21.3	66.8	-	168.7
TPU_Cu F	−19.9	78.2	-	178.3
TPU_Cu P	−19.8	62.2	-	176.4

**Table 4:** Optical density of the culture media suspensions after 38 h of incubation with the filaments and 3D-printed specimens.

Materials		Optical Density (MacFarland Scale)		
		*E. coli*	*P. aeruginosa*	*S. aureus*
Filament	PLA	2.26 ± 0.10	2.35 ± 0.08	2.10 ± 0.06
	PLA_Cu	0.05 ± 0.02	0.07 ± 0.01	0.02 ± 0.01
	TPU	2.53 ± 0.08	2.30 ± 0.07	2.00 ± 0.09
	TPU_Cu	0.04 ± 0.01	0.05 ± 0.02	0.03 ± 0.02
3D-Printed filters	PLA_Cu Linear 100%	1.10 ± 0.05	1.20 ± 0.07	1.00 ± 0.05
	PLA_Cu Linear 75%	0.98 ± 0.06	1.00 ± 0.05	0.91 ± 0.07
	PLA_Cu Linear 25%	0.75 ± 0.10	0.84 ± 0.11	0.70 ± 0.08
	TPU_Cu Linear 100%	1.05 ± 0.05	1.10 ± 0.53	1.00 ± 0.02
	TPU_Cu Linear 75%	0.96 ± 0.07	1.00 ± 0.02	0.89 ± 0.08
	TPU_Cu Linear 25%	0.66 ± 0.10	0.74 ± 0.10	0.60 ± 0.11
	PLA_Cu Hexagonal 75%	0.93 ± 0.06	1.05 ± 0.07	0.93 ± 0.04
	PLA_Cu Hexagonal 25%	0.70 ± 0.10	0.74 ± 0.09	0.72 ± 0.05
	TPU_Cu Hexagonal 75%	0.90 ± 0.03	0.99 ± 0.07	0.90 ± 0.04
	TPU_Cu Hexagonal 25%	0.62 ± 0.10	0.75 ± 0.13	0.61 ± 0.12
	PLA_Cu Triangular 75%	0.91 ± 0.06	1.07 ± 0.05	0.90 ± 0.07
	PLA_Cu Triangular 25%	0.69 ± 0.12	0.79 ± 0.10	0.75 ± 0.06
	TPU_Cu Triangular 75%	0.87 ± 0.05	1.00 ± 0.08	0.93 ± 0.06
	TPU_Cu Triangular 25%	0.59 ± 0.03	0.8 ± 0.09	0.58 ± 0.04

## Data Availability

The original contributions presented in this study are included in the article. Further inquiries can be directed to the corresponding author.
